# Drugs, distrust and dialogue –a focus group study with Swedish GPs on discharge summary use in primary care

**DOI:** 10.1186/s12875-018-0804-8

**Published:** 2018-07-25

**Authors:** Gabriella Caleres, Eva Lena Strandberg, Åsa Bondesson, Patrik Midlöv, Sara Modig

**Affiliations:** 1 Department of Clinical Sciences in Malmö/Family Medicine, Center for Primary Health Care Research, Lund University, Jan Waldenströms gata 35, SE-205 02 Malmö, Sweden; 2 Department of Medicines Management and Informatics in Skåne County, Kristianstad, Sweden

## Abstract

**Background:**

Discharge summary with medication report effectively counteracts drug-related problems due to insufficient information transfer in care transitions. The benefits of the discharge summary may be lost if it is not adequately used, and factors affecting optimal use by the GP are of interest. Since the views of Swedish GPs are unexplored, this study aimed to explore and understand GPs experiences, perceptions and feelings regarding the use of the discharge summary with medication report.

**Method:**

This qualitative study was based on four focus group discussion with 18 GPs and resident physicians in family medicine which were performed in 2016 and 2017. A semi-structured interview guide was used. The interviews were transcribed verbatim and analysed using qualitative content analysis.

**Results:**

The analysis resulted in three final main themes: “Importance of the discharge summary”, “Role of the GP” and “Create dialogue” with six categories; “Benefits for the GP and perceived benefits for the patient”, “GP use of the information”, “Significance of different documents”, “Spider in the web”, “Terminus/End station” and “Improved information transfer in care transitions”. Overall, the participants described clear benefits with the discharge summary when accurate although perceived deficiencies were also quite rife.

**Conclusion:**

The GPs experiences and views of the discharge summary revealed clear benefits regarding mainly medication information, awareness of any plans as well as shared knowledge with the patient. However, perceived deficiencies of the discharge summary affected its use by the GP and enhanced communication was called for.

## Background

The World Health Organization (WHO) recognizes patient safety as a ‘serious global public health issue’ [1]. As many as 10% of patients are harmed as a result of inadequate hospital care; the costs of the deficits in patient safety are comprehensive [1]. Care transitions are particularly risky, and adverse health outcomes and re-admissions are common particularly among older people after hospital discharge [2, 3].

The National Board of Health and Welfare in Sweden points out the need for routines concerning medical information transfer in care transitions in order to ensure fast and accurate updates on drug changes during hospitalization as well as adequate follow-up in primary care [4]. Current guidelines state that a *medical case history* as well as a discharge summary with medication report should be transferred at hospital discharge [5]. The *medical case history* is a detailed document on the hospital stay, describing the cause of admission, course of medical care as well as any follow-up plans, without any medication information [6]. The discharge summary contains similar information but in a briefer form, comprehensible to a layman [7]. The discharge summary also entails a medication report, which summarizes which changes in medication were made and why, as well as a medication list, which should be followed-up by the patient’s GP [8]. The *medical case history* is sent to the general practitioner (GP), while the discharge summary is both sent to the GP and given to the patient*.* However, lacking information transfer in care transitions is common [9–11]. This may complicate the work for the GPs who require accurate and fast information on actual medications and follow-up in order to maintain good continuity of care [9, 12, 13]. In a systematic review of qualitative studies, it was noted that lacking communication with many prescribers involved and unsatisfactory information transfer at care transitions were cited as contributing to information deficit identified as a barrier to minimizing potentially inappropriate medications [14].

Elderly patients in particular commonly experience medication errors in care transitions [15]. Many medication errors are potentially harmful [16] and may lead to preventable adverse drug events (ADEs) [17]. However, many adverse drug reactions are likely to be preventable [4, 18, 19]. Discharge summaries reduce medication errors and health care consumption [20, 21]. The benefits of the discharge summary may be lost if it is not adequately transferred and received. Primary care has a clear follow-up responsibility, and insights concerning obstacles and possibilities for optimal use of the discharge summary would be of value. The views of Swedish GPs are, as yet, unexplored.

The aim of this study was to explore and understand GPs experiences, perceptions and feelings of the use of the discharge summary with accompanying medication report.

## Methods

### Study design

The methodological orientation of this study was qualitative content analysis, which entails organizing and subjectively interpreting text data [22, 23]. Data were collected via focus group discussions; a suitable method to explore experiences and views of a group that has shared knowledge and experiences [24, 25]. Focus groups are also suitable to study perceptions, thoughts and feelings as well as factors affecting behavior [24]. Focus groups consist of a smaller group of people; ideally few enough to let everyone share but with enough participants to generate width [24]. The qualitative data emerging from the focus group discussion is used to understand a specific subject, which in this case was the GPs’ experiences of and views regarding the discharge summary [24].

### Setting and participants

This study was conducted in Skane, a region in the south of Sweden, that has just over 150 primary care units and over 800 General Practitioners (GPs) [26] and where 1.3 million (13%) of the Swedish population live [27]. Both the hospitals and primary care units have separate electronic medical records.

Participants were GPs and resident physicians in family medicine, i.e. health care professionals joined by their common experiences of working in primary care. Focus groups were recruited from pre-existing groups (so called FQ-groups; an acronym for training and quality in Swedish), originally formed to ensure in-service training and quality development in family medicine by the professional and scientific college of GPs in Sweden (SFAM) [28]. GPs are in general members of FQ groups by interest. In this region, all resident physicians in family medicine are referred to a FQ group from start. The use of pre-existing groups was expected to enable discussions due to well-established interplay. Furthermore, FQ-groups include men and women of different ages with varying lengths of time in the profession. Three focus group discussions were initially conducted with preparation done for a fourth group. To reach saturation [24], the fourth focus group discussion was also conducted, in which nothing new emerged. Altogether, 18 women and five men working in both rural and urban areas participated in the focus groups. A total of 14 of the participants were GPs, and nine were resident physicians in family medicine.

### Procedure

The focus group discussions took place between December 2016 and February 2017. The selection of groups was mainly purposive and strategical to ensure variation of gender, age, professional experience and working environment. In one case, two group leaders were approached by email but only one responded. Otherwise, no participants refused to collaborate. Two groups consisted of only GPs, one group consisted of only resident physicians and one group was mixed. For clarity, all participants will be referred to as GPs. A letter of information about the study and the research method involved was sent to the participants before the focus group discussion.

A semi-structured interview guide in Swedish, developed by ELS and GC, was used. A semi-structured guide describes the topics to be covered, with proposed questions, but how closely the interviewer must stick to the guide varies from study to study [29]. The focus of the discussion in this study was the discharge summary with medication report, and its use in primary care. The participants were asked to discuss their experiences of the discharge summary and its use, and thereafter about the medication report. The interview guide also involved a checklist that was used to ensure that relevant issues were touched upon. If not, they were introduced into the discussion. The interview guide was revised between the focus group discussions when a new issue emerged. For example, the patient’s perspective and access to hospital medical records was gradually added to the checklist.

Two of the authors (GC and ELS) conducted the focus group discussions. The discussions were moderated by GC, a resident physician working in family medicine but with no previous experience of qualitative research. ELS, an associate professor in community medicine with comprehensive experience of qualitative research and thorough knowledge of primary care, assisted the focus group discussions and took notes. Afterwards, a debriefing took place for feedback and further development of the conduct of the focus group discussions [24]. Two of the focus group discussions took place in the meeting rooms of two different primary care units. One focus group discussion took place in an administrative building that was connected with the hospital after a lecture for resident physicians. One focus group discussion was conducted in the home of one FQ-group member, which was where they normally met.

The discussions were audio recorded and transcribed verbatim.

### Ethical considerations

The Regional Ethical Review board (EPN; Etikprovningsnamnden/Ethical Review Board), Lund approved the project (registration number 278/2016, protocol 2016/9). The Regional Ethical Review board in Lund (whose secretariat is situated at Lund University) is an independent authority, supervised by the Ombudsman and Attorney General of Sweden. Written informed consent was obtained from all participants. Participation was voluntary. The collected data cannot be linked to any participant.

### Analysis

A qualitative content analysis of the manifest and latent content was performed [23, 25]. First, the interviews were listened to and read through several times by GC, to gain a sense of the whole. Meaning units were identified and condensed in collaboration with ELS, who had also read the interviews. Initially, the material was roughly classified into tentative themes, a process inspired by Malterud [25]. Thereafter, it suited the material better to continue with content analysis guided by Graneheim and Lundman [23]. The statements were preliminary categorized. Thereafter, GC, ELS and SM (a GP with many years of clinical experience as well as having conducted qualitative research, who had also read the interviews independently) elaborated this material into sub-categories as the categories were found to have different levels. Categories were finalized, and eventually led to three overall themes after careful discussion to reach agreement. The analysis was confirmed by the other two authors (PM and ÅB). An example of the analytical process is shown in Table 1*.*

**Table 1:** Example of the analytical process

Meaning unit	Sub-category	Category	Theme
Sometimes I feel vague... When information is missing, I feel I have to decide, because I hold the *main responsibility* for the patient, should she take this yes or no. (FG 1) It would be good, or one would wish to be able to see the patient quite rapidly after discharge, to check quickly to make it right. (FG 4) After drug changes they forget to correct the multidose dispensed drugs^2^. You have to change this very often, and they often make mistakes, in every other case. And this takes extra time for us to correct. (FG 4)	*Main responsibility* Follow-up Clean up	Terminus/End station	Role of the GP

^2^Machine-dispensed disposable sachets in which medications are packaged according to the time of administration 30. Bergman A, Olsson J, Carlsten A, Waern M, Fastbom J: Evaluation of the quality of drug therapy among elderly patients in nursing homes. Scand J Prim Health Care 2007, 25(1):9–14

From our preconceptions and experiences from working in and studying primary care, we mainly relate our findings to the concept patient safety for elderly in care transitions.

## Results

**Fig. 1:**
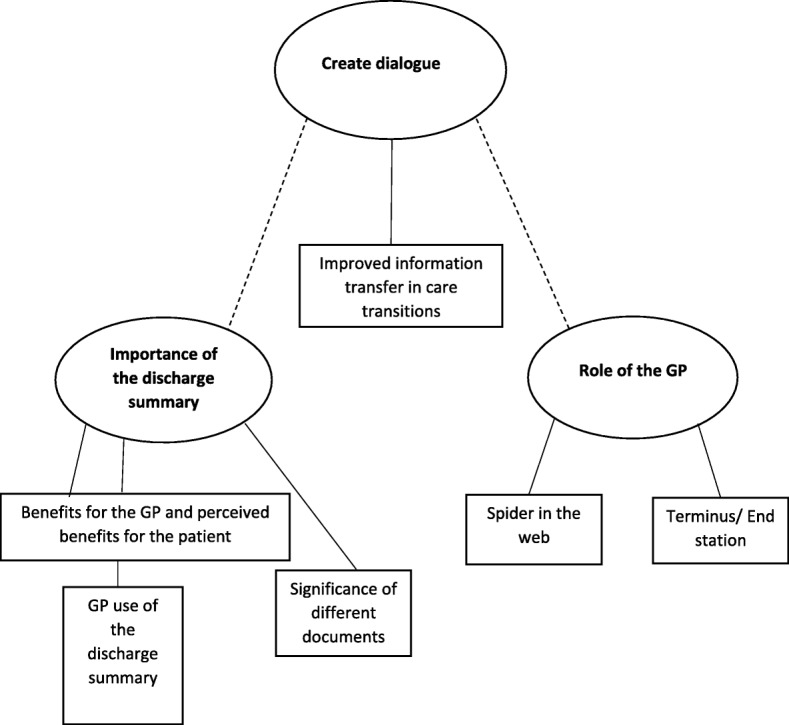
Final three themes with categories

The tentative themes initially seen in the text that formed the basis for further analysis were “benefits of the discharge summary”, “disadvantages of the discharge summary”, “spider in the web and compensating strategies”, “desires and solutions” and “insecurity”. Further analysis of the material resulted in three final main themes: “Importance of the discharge summary”, “Role of the GP” and “Create dialogue” with six categories (Fig. 1). Themes and categories are described below, with subcategories in *italics* built into the text.

### Importance of the discharge summary

The advantages as well as the inadequacies of the discharge summary underlaid the theme ‘importance of the discharge summary’.

Benefits for the GP and perceived benefits for the patient.

This category is exemplified in Table 2. Many viewed the discharge summary as very useful when accurate, above all when it came to receiving an *updated medication list.* A clear and unanimous medication report and medication list were undoubtedly helpful.

**Table 2:** Category Benefit for the GP and perceived benefits for the patient and examples of quotations of some of its *sub-categories*. (Theme 1)

*Accurate medication list* *“*If it works well, they’re well written and updated regarding medications, they’re great, of great help.” (FG 1) “You check the medication list at discharge with ours and make corrections. When the patient revisits, you check again, is this correct, sometimes they quit a pill for some reason…and then you update it.” (FG 3) *To know what the patients know* “I can assure... I know the patient knows what’s been said (in the discharge summary), if I get it from the *medical case history* I don’t know how much of this the patient has perceived from their hospitalization, but the discharge summary at least, I know the patient has read…perhaps, or at least received. What is in the discharge summary, I feel safe that we know the same things.” (FG 2)	

“If it clearly says ‘you were prescribed the following drugs during hospitalization’...then I assume the patient knows this is the new drug and I also add it to the medication list.” (FG 2).

When the discharge summary felt reliable, the medication list was often described as its main advantage.

Knowledge of any *plans for the patients* was also of benefit to the GPs, as well as the patients. Well-informed patients know where to turn in case of any unforeseen events, and likewise the GPs benefit from knowing where the patients should be referred to. The discharge summary also clarifies for the GPs what is expected from them and which follow-up is planned elsewhere, as noted here:

“The advantage for us when it exists and it is good, is that a well-informed patient who can follow the plan simplifies for us, who knows when something deviates and can act. In that way... the chance of things going right increases when the patient is informed.” (FG 3).

Furthermore, to *know what the patients knew* about their hospitalization was considered very valuable. This facilitated further communication with the patients. It is also easier for the GPs when the patients understand what has happened during the hospitalization. Together, these factors implied a sense of security and a better chance to ‘get things right’.

Some viewed the discharge summary of greatest use for the patients and their relatives, and described it as mainly *the patient’s document*. Sometimes, this implied the discharge summary was of less use for the GPs, as exemplified here:

“But on the other hand I believe it is great for the patient, and for their relatives, that it exists, even if I don’t use it a lot.” (FG 2).

A notion that the discharge summary was originally constructed for the patients rather than health care professionals was also expressed.

Further, it was agreed that the patients obviously also benefit from *an accurate medication list*. Not least when the GPs needed to communicate with nurses in municipality care. If these nurses requested an accurate medication list, the GPs would compare available medication lists and fit them together. The patients also benefit from the GPs detecting and correcting any errors and making a decision on the medication list, as noted here:

“Perhaps I at least look it through to see if anything is odd… very different or added as compared to before” (FG 4).

The benefits for the patients of *shared knowledge* and clear allocation of responsibilities were also highlighted. It helps when the patients, their families as well as all caregivers involved know what has been done and who is going to do what in the future. As one respondent noted:

“I’m thinking of relatives also, if you imagine an elderly patient with dementia who has no clue what happened, it can simplify for relatives to see what has been done during hospitalization. And the home nursing staff. Staff at the retirement home.” (FG 3)

#### GP use of the discharge summary

The GPs expressed good use of the discharge summaries mainly regarding *information on medication* as noted above. Detailed information was requested, for example clear motivation for adding or withdrawing a drug. This information was used to update the medication list in primary care electronic medical records, or to do a medication reconciliation. Sometimes, this was the sole area of use.

“For us, only the medications are interesting.” (FG 3).

Apart from that, a widespread feeling of *distrust* was revealed. Basically, this originated in the uneven quality of the discharge summaries, but also from the GPs’ own previous experiences of having written discharge summaries themselves. Insecurity regarding the patient’s ordinary medications at hospital admission and how to handle this was described.

The quality was also considered to differ depending on who wrote it (e.g. a medical intern or a chief physician) and where it was written, as stated here:

“If the patient has received care in the orthopaedic or surgical department, I reckon these are the drugs the patient has been given during the hospital stay, nothing else, it says nothing about the big picture.” (FG 2).

It was also suggested that the physician who had written the discharge summary sometimes lacked knowledge about the patient, as noted in the following quote:

“I think it’s good when it is there.

The idea is good, I think.

But that it’s often made quickly by someone who hasn’t met the patient before, I believe.” (FG 3).

Distrust could also stem from a discrepancy in regard to the medication list in the primary care electronic record, in which case the medication list in the discharge summary was sometimes viewed as ‘useless’. A sense of resignation was also expressed, with the vast amount of uncertainties regarding medication, affecting the chance of an accurate medication list. One participant expressed that she would not miss the discharge summary if it wasn’t transferred from the hospital since she ‘didn’t trust it (i.e. the discharge summary)’.

A common reason for not using the discharge summary optimally was cited as *lack of time*. There was sometimes a sense of guilty conscience - you know that you should use the discharge summary to update the medication list and aspire to do it - but the workload is simply too overwhelming as expressed here:

“I don’t always have time, sometimes the (drug) lists are so dreadfully long that I sort of…that it isn’t done until the patients next visit.” (FG 1).

The significance of different documents.

The *medical case history* was often described as superior to the discharge summary, since it is strictly medical. A couple of participants saw very limited use of the discharge summary in the light of the *medical case history,* even though most were not as categorical as this participant:

“I haven’t understood at all why the discharge summary was introduced, except for the benefit of the patient of course, but why it should be of benefit to health professionals... I don’t understand, since we have the *medical case history*” (FG 3).

Further, for some, the main raison d’être of the discharge summary was the medication list, which is generally not included in the *medical case history*.

The need of a was repeatedly emphasized for the GPs to take clear responsibility for the patients. This was described as a requirement for any follow-up, sometimes implying a way to acquit oneself from responsibility as seen in this quotation:

“They are obliged to send a *referral* if we are to follow-up, at least that’s what I’ve been taught.

If we should actively follow-up, that is if we are going to take any action.

Then I sort of read...the discharge summary and *medical case history,* I screen in case it’s been missed, but... well, it’s not my fault in that case, I think.” (FG 2).

This may also be interpreted as a way to protect oneself from everything that is passed on.

The wish for *a common medical record and medication list* in hospital and primary care was also a major point of discussion in all focus group discussions, although the question of how to handle such vast amounts of information was also raised.

A common medical record.

Yes, that would be golden.

Pros and cons, on second thought. Because it would be very, very much information. (FG 4).

However, a common medication list was a general desire, and there was a strong belief this would prevent many problems regarding medications due to lacking information transfer in care transitions.

### Role of the GP

In this theme, ‘the role of the GP’ was expressed as difficult and challenging but also rewarding, since it entails holding the overall responsibility for the patient and pulling all strings together. This entails receiving and using the discharge summary.

#### Spider in the web

This category is exemplified in Table 3. The role of the GP was commonly described as the spider in the web and viewed as *a basic condition of being a GP.* This notion is a central part of the job, and also something the patients and other caregivers expects from primary care, whether one likes it or not. As two respondents noted:

“Primary care holds the overall responsibility...they expect that from us, for us to be the spider in the web and make sure everything is done and done in the right way. The patients also believe...we have control.” (FG 4).

“I haven’t thought about, whether it’s boring or fun, because it’s part of my job…the GP is the spider in the web somehow, we can think what we want, we have to do it anyway.” (FG 3).

**Table 3:** Category Spider in the web and examples of quotations of some of its *sub-categories.* (Theme 2)

*Patient’s interpreter* “Sometimes they (patients) haven’t got the information from the hospital, then they wonder. Most often they come to us and ask. Either they didn’t get it or they didn’t understand it.” (FG 4) *Detectives* “I look in the hospital electronic medical records, to see what the admission record says, or where else my drug disappeared along the way. If it is not in the admission record, they have forgotten to tell they’re taking that blood pressure pill or so. That’s how I use it, more to compare. You have to be somewhat of a detective there, to see why it disappeared.” (FG 3) *Consistent information to the patients* “Perhaps we should be more careful in handing out medication lists to patients, at every visit, and note when we change something, but then there are other operators that we can’t wrap our heads around.” (FG 4)	

Being the heart of the care chain also meant being the *patient’s interpreter* in regard to their care, due to lack of or unintelligible information. To help the patients understand and guide them through their care process is a vital part of the role of the GP. Further, the GPs also described themselves as *detectives* searching in all possible ways for correct information, which was also a basic condition of their role. This was often described as dull, challenging and time-consuming, although the occasional participant found being ‘the liaison office’ rather rewarding.

Several participants had obtained access to the hospital electronic medical record and viewed this as a prerequisite to get access to complete and correct information.

“There is no balance in this, we in primary care we fight tooth and nail in all different systems to…to find the truth somehow. We have to work with this all the time, it is a prerequisite for us to be able to do our job, to be everywhere.” (FG 2).

Even though this meant duplication of effort, it was used as a sort of safety net. Nevertheless, others abstained from using the hospital medical records due to the limitless amount of information, and viewed using the hospital records as making things too ‘messy’.

Finally, the need of *consistent information to the patient* from all caregivers was discussed. If, for example, every caregiver the patients met would inform them carefully about their medications, this would likely prevent some mistakes and misunderstandings and also enable the patients to assist in future drug-related communication.

#### Terminus/end station

This category is exemplified in Table 1, and entailed the notion of holding the *main responsibility* for the patient, sometimes reluctantly. A feeling of being the last resort was expressed. Holding the *main responsibility* went without saying but entailed mixed emotions.

“I feel it’s quite hard sometimes actually.

Yes, sometimes it would’ve been very nice to be able to say: Go to your GP!

Yes, it feels like we are the last resort.” (FG 4).

A wish for improved primary care follow-up of the patient, was noted, not the least in regard to the medication list. Being able to see the patient on a revisit quite soon after discharge, or having someone else phoning the patient (a nurse, for instance) was believed to prevent errors.

There were also feelings of frustration coming from having to ‘clean up the mess’ coming from, for example, lacking updates of Multidose dispensing in the hospital but also due to incomplete information transfer documents (Multidose drug dispensing means machine-dispensed disposable sachets in which medications are packaged according to the time of administration [30]). One participant reacted particularly strongly to the feeling that some of the hospital physicians seemed to assume the GPs would correct their mistakes. Another participant who had recently worked in the hospital environment was more forgiving.

### To create dialogue

This theme mainly concerned different ways to enhance the communication concerning the patient between hospital and primary care. The theme is closely related to and have a bearing on the other two themes, without being superior to them.

#### Improved information transfer in care transitions

This category is exemplified in Table 4 where a wish for enhanced dialogue was expressed. The possibility for the GPs to *participate in the discharge* of the patients was discussed. However, this was not considered feasible in reality. Still, the notion was this might prevent many mistakes. One GP had surprisingly received a telephone call from the hospital physician before her patient was discharged, which she said gave her a strong feeling of importance. The possibility to receive the information in the discharge summary prior to discharge was also discussed. To *formally acknowledge the discharge summary* before taking over the medical responsibility would allow for correction of mistakes and a chance to sort out potential errors before discharge. This would increase the trust in the discharge summary, as noted here:

“If we could get this information in SVPL^1^ instead, and to use that information before actively taking over the medical ...then you have to make that time at least...and think through. Perhaps you trust what comes later more then.” (FG 4).

Similarly, the quality of the discharge summary would be viewed as higher if *information regarding any pharmacist medication review* would be present.

**Table 4:** Category Improved information transfer in care transitions and examples of quotations of some of its *sub-categories.* (Theme 3)

*GPs participation in the discharge of the patients* “Ideal would be for us to *participate in the discharge* and make corrections there really, but there is no chance in the world that would be possible.” (FG 4) *Information regarding any pharmacist medication review* “It would be good if we had that information, that a pharmacist medication review was done during hospitalization, it would be valuable. It enhances the quality of the medication list in the discharge summary. Considerably, I believe. This information is important.” (FG 3)	

## Discussion

The main results of this study are described by three final themes; “Importance of the discharge summary”, “Role of the GP” and “Create dialogue”. Overall, the participants described clear benefits with the discharge summary when accurate, mainly regarding medication information, awareness of any plans as well as shared knowledge with the patient. However, perceived deficiencies of the discharge summaries were also quite salient, and improved communication was called for to give the GPs optimal conditions in their role as the spider in the web.

Based on their experiences and views of the discharge summary, our participants saw room for improvement mainly through enhanced communication, in accordance with hospitalists and GPs in the study by Jones et al. who wished for improved direct access to each other to improve care coordination in care transitions [31]. Greater efforts regarding coordination care for high-risk patients were also proposed [31], which is in line with a new law in Sweden that came into effect from 1 January 2018 [32]. This law aims to encourage cooperation between all caregivers at patient discharge, with increased primary care participation in discharge planning and follow-up [32], relating well to the desires of our participants. Since they nearly saw creating a dialogue as utopian, the rapid implementation of this law is quite remarkable. The chance to participate more extensively in discharge planning and follow-up may also provide the GPs with a sense of empowerment and occupational pride.

A major benefit of the discharge summary was the medication information according to our participants, which is in line with the views of GPs in previous studies by Karapinar [9], Robelia [32] and also by Yemm et al., who compared GPs and hospital junior doctors’ opinions of the discharge summary [13]. Accuracy was most important for both groups [13], which is in line with our study. Further, our participants also reported the discharge summary helped improve their communication with the patients through shared knowledge of what had happened during hospitalization as well as of any follow-up plans. This was viewed as important for patient safety thus possibly helping to improve care transitions and reduce health care consumption. These important aspects were overlooked by the few participants regarding the discharge summary as solely the patient’s document. Few discharge summary studies embrace the patient’s perspective, but in a Dutch study, hospital physicians, GPs as well as patients all experienced deficits in information transfer at hospital discharge while GPs in particular experienced poor coordination of responsibilities [33].

Furthermore, a great deal of distrust was expressed by our participants, often due to poor discharge summary quality. Deficient discharge information is previously commonly noted, mainly regarding medication information [11, 12, 32, 34] and follow-up plans [10, 12]. Such shortcomings may affect the quality of care at follow-up [12] and dissatisfy the GPs [12, 13]. Adequate medication information counteracts medication errors at discharge [20]. Hence, the patient also benefits from an updated medication list as noted in our study, which also entails the GP detecting and correcting of any errors. In light of this, an inadequate discharge summary might be better than no information at all, at least offering the possibility to detect any errors and inform the patient.

Distrust was also related to by whom and where the discharge summary was written. However, an Australian study showed no impact of physician medical training level on discharge document medication error rate [35], while medical patients in another study had more errors than surgical patients [16]. GPs and hospital doctors’ opinions on the discharge summary content may also differ; in the study by Yemm et al. considerably fewer hospital junior doctors ranked medication changes of great importance [13]. Also, many felt the training received for writing discharge summaries was insufficient [13]. Hospital doctors in the region of our study receive no formal discharge summary writing training, although minor educational efforts were offered within a prior quality improvement project [36]. Conversely, our participants viewed the discharge summary quality as higher when *information regarding any pharmacist medication review* was present, relating well to the GPs in a study by Karapinar et al., who largely appreciated pharmacotherapeutic advice [9].

In addition to distrust, our participants pointed out lack of time as an important reason for not using the discharge summary optimally, which is in line with the GPs in a report from the National Board of Health and Welfare in Sweden who noted lack of time as a critical factor in caring for the elderly, negatively affecting follow-up [4]. Lack of time may reflect upon prioritization; time will most likely be spent on work that is perceived as meaningful. Hence, this may differ between participants with positive and negative experiences and views of the discharge summary respectively, which stresses the importance of an accurate discharge summary worth being prioritized. However, keeping medication lists accurate is a fundamental responsibility for any physician, essential for adequate patient assessment and patient safety. Further, as noted in the before mentioned report, time is a necessary resource to enable adequate follow-up and medication reconciliation, and the direction must allocate sufficient time for this issue to signal its importance and priority [4].

Lack of time may also influence the demand for a *referral* for any follow-up made by many participants. With an increasingly heavy work burden, this may be a way to protect one-self. However, the patient’s best interest and safety is a vital responsibility shared by all physicians. Also, the Swedish National Board of Health and Welfare clearly states that the patients’ ordinary physician (i.e. GP) is responsible for medication follow-up after discharge with a medication report [8]. In a study by Jones et al., the GPs wished for this to be clarified via discharge summaries or direct communication [31].

In our study, the GPs were described to have a crucial, coordinating role. Apparently, the discharge summary was only one of many ways the GPs used to collect information on the hospitalization. Relevant information was sought via different sources, such as by gaining access to the hospital medical electronic records not normally accessible to the GPs, clearly suggesting failure of the information transfer. Indeed, common medical records is often viewed as a way to improve communication [4, 31], but must be kept accurate to be of value [4, 37]. *A common medical record and medication list* was also a general wish for the future in our study, which however does not eliminate the responsibility to work for well-functioning care transitions in the meantime.

Further, the GPs described having to ‘clean up’ i.e. performing duties ought to have been completed by hospital physicians. Such tasks may contribute to an excessive workload for the GPs. The increasing and unsustainable workload of British GPs was perceived as partly due to lacking information transfer in a previous qualitative study [38]. Deficient information transfer with discharge summaries was also noted in the report from the National Board of Health and Welfare [4]. Adequate discharge information is necessary since the GPs’ role, according to our participants, also entailed holding *main responsibility* and explaining certain aspects of the care to the patients, to avoid misunderstandings, needless care-seeking and follow-up issues. Being the spider in the web was frustrating at times, but a basic and often enjoyable condition of being a GP. To maintain job satisfaction and counteract an overwhelming workload for the GPs, information transfer with discharge summary needs to be accurate not to merely be viewed as a burden rather than of any help.

### Methodological considerations

To achieve trustworthiness in qualitative research, several aspects must be considered [23]. Regarding credibility, the participants in our study had different experiences, gender and work environments although they were all general practitioners or training to be, contributing to variety and richness of the collected data. Further, focus group discussions are commonly described as an appropriate method to explore experiences and views [24]. However, using pre-existing groups may imply some challenges. For instance, the discussion might be affected if the group members know each other too well. Established negative patterns may influence the discussion, such as one person being dominant and not letting all voices be heard. However, this was not commonly noted in our study. When needed, the moderator invited more low-key participants to join in.

Triangulation was also aspired. Two of the authors discussed the selection of meaning units to reach consensus. Seeking agreement was also done in the subsequent analysis, where three of the authors with different experiences and background had an ongoing dialogue to confirm the results and its interpretation. The analysis was further confirmed by the other authors. As for transferability, these experiences may be common in similar conditions, although the aim of qualitative studies is not primarily to generalize. Nevertheless, the views expressed are those of our participants, and could have been different with other participants, although saturation was reached i.e. nothing new emerged in the final groups. Researcher reflexivity is also central to research quality [25, 39]. Our overall preunderstanding and experiences from working in and studying primary care as well as conducting qualitative research naturally affected our choice of research perspective and to some extent our interpretation of the results. Our knowledge in this area was truly useful in conducting the focus group discussions, and our different perspectives prevented our preconceptions from negatively affecting the research process and helped us to a fuller understanding of our data.

### Future research

Many participants expressed distrust originating from experiences of deficient discharge summaries, mainly regarding the medication information which was of utmost importance to the GPs. Therefore, exploring and understanding factors affecting the quality of the medication information in the discharge summary would be of interest and may allow for targeted actions of improvement, increasing benefit for and utilization by the GPs. Further exploration of the patient’s perspective could also be valuable, as well as the views of the hospital doctors who write the discharge summaries.

## Conclusion

Exploring the GPs experiences and views of the discharge summary revealed clear benefits primarily regarding medication information and follow-up, essential to patient safety. However, the quality of the discharge summary was not perceived as entirely satisfactory which greatly affected its use by the GP; the spider in the web in need of full access to accurate patient information. Enhanced communication was called for. The distrust many GPs expressed poses a great challenge, and looking into the accuracy of the discharge summary from a primary care point of view would be a fruitful next step.

## Footnotes

1
*An electronic system regarding the patient’s current status, used mainly by nurses at the hospital and in municipality care for planning care transitions, but also requires filling in the name of the patient’s GP.*
